# Comprehensive Screening and Identification of Phillyrin Metabolites in Rats Based on UHPLC-Q-Exactive Mass Spectrometry Combined with Multi-Channel Data Mining

**DOI:** 10.1155/2020/8274193

**Published:** 2020-06-24

**Authors:** Beibei Ma, Jiameng Li, Tianyu Lou, Yaoyue Liang, Chenxiao Wang, Ruiji Li, Tingting Wang, Jinhui Liu, Yudong Guo, Zhibin Wang, Jing Wang

**Affiliations:** ^1^School of Chinese Pharmacy, Beijing University of Chinese Medicine, Beijing, China; ^2^Beijing Institute for Drug Control, Beijing, China; ^3^Beijing Research Institution of Chinese Medicine, Beijing University of Chinese Medicine, Beijing, China

## Abstract

Phillyrin, a well-known bisepoxylignan, has been shown to have many critical pharmacological activities. In this study, a novel strategy for the extensive acquisition and use of data was established based on UHPLC-Q-Exactive mass spectrometry to analyze and identify the *in vivo* metabolites of phillyrin and to elucidate the *in vivo* metabolic pathways of phillyrin. Among them, the generation of data sets was mainly due to multichannel data mining methods, such as high extracted ion chromatogram (HEIC), diagnostic product ion (DPI), and neutral loss filtering (NLF). A total of 60 metabolites (including the prototype compound) were identified in positive and negative ion modes based on intuitive and useful data such as the standard's cleavage rule, accurate molecular mass, and chromatographic retention time. The results showed that a series of biological reactions of phillyrin *in vivo* mainly included methylation, hydroxylation, hydrogenation, sulfonation, glucuronidation, demethylation, and dehydrogenation and their composite reactions. In summary, this study not only comprehensively explained the *in vivo* metabolism of phillyrin, but also proposed an effective strategy to quickly analyze and identify the metabolites of natural pharmaceutical ingredients in nature.

## 1. Introduction

Phillyrin, as an essential bisepoxylignan, is the main component of *Forsythia suspensa* (Thunb.) Vahl in plants belongs to the family Oleaceae [1–3]. Modern pharmacological research has shown that phillyrin not only has potent biological activity, but also plays a huge role that cannot be ignored in resisting diseases and maintaining human health, such as inhibiting inflammatory response, antiviral, antioxidative stress, and anticell apoptosis [4–8]. Phillyrin has been reported to improve insulin resistance in the body [9, 10], and it can also reduce the weight of obese mice through specific pathways [11]. Besides, phillyrin can decrease the formation and function of osteoclasts and prevent LPS-induced osteolysis in mice [12]. However, there are still many deficiencies in the comprehensive research on phillyrin metabolism *in vivo*. Therefore, revealing the metabolites and metabolic pathways of phillyrin is of great significance for its further development and utilization.

As we all know, liquid chromatography-mass spectrometry (LC-MS) combines the high separation power of liquid chromatography with the forceful qualitative and quantitative ability of mass spectrometry, which has the advantages of high sensitivity, high selectivity, and rich structural information [13–15]. UHPLC-Q-Exactive MS (ultra high-performance liquid chromatography coupled with hybrid quadrupole-Orbitrap mass spectrometry) can thoroughly and extensively obtain the structural information of compounds by using the functions of positive and negative ion scanning mode, full scanning, and automatic triggering of secondary mass spectrometry scanning. The superior resolution based on Orbitrap technology can quickly achieve the measurement of high-accuracy mass. Therefore, it can identify and confirm small molecule compounds in mixtures, is a powerful tool for analyzing complex compound systems, and has been extensively used in compound identification and screening [16–18]. By obtaining sufficient fragment information and analyzing composition information of a compound online, the molecular weight of the compound can be determined, and the possible molecular structure can be inferred based on the primary, secondary, and even multilevel mass spectral information [19–21]. In consequence, it is imperative to form a comprehensive and authoritative data set in the data processing process. Data mining methods reported in recent years have emerged endlessly, mainly including the following categories: mass defect filter, extracted ion chromatogram, diagnostic product ion (DPI), neutral loss filtering (NLF), and isotope pattern filtering [22–27].

In this paper, we have established a new strategy that combined UHPLC-Q-Exactive MS with multiple data mining analysis methods. Due to its slight detection limit and narrow deviation range, it is feasible to analyze and identify metabolites in rats for oral administration of phillyrin. Based on this study, we further proposed the *in vivo* metabolic pathways of phillyrin, which made up for the shortcomings of the current insufficient research on phillyrin metabolism and was conducive to revealing the *in vivo* mechanism of many pharmacological activities of phillyrin.

## 2. Materials and Methods

Based on the previous research results of our research group, we adopted the experimental methods and sample processing procedures of our predecessors [28, 29].

### 2.1. Chemicals and Reagents

The reference standard of phillyrin was purchased from Chengdu Must Biotechnology Co. Ltd. (Sichuan, China). After HPLC-UV analysis, the purity of phillyrin was not less than 98%. Acetonitrile, methanol, and formic acid (HPLC grade) used in the mobile phase were provided by Fisher Scientific (Fair Lawn, NJ, USA). C18-low solid-phase extraction (SPE) cartridges (3 L/60 g) for biological sample pretreatment were obtained from Waters (Milford, MA, USA). Ultrapure water was freshly prepared using the Milli-Q Gradient Å 10 water purification system (Millipore, Billerica, MA, USA). Moreover, other reagents and solvents met the requirements of analytical experiments in Beijing Chemical Works (Beijing, China).

### 2.2. Animals and Drug Administration

Sixteen male SD rats weighing 200–220 g were purchased from Beijing Weitong Lihua Experimental Animals Company (Beijing, China). All animals were kept under specific environmental conditions (temperature 22 ± 1°C, humidity 60 ± 10%, 12-hour day and night change), with free access to food and water for one week. The rats were then randomly divided into Drug Group (for experimental urine, plasma and feces, *n* = 8) and Control Group (for blank urine, plasma, and feces, *n* = 8). The reference standard of phillyrin was suspended in 0.5% sodium carboxymethyl cellulose (CMC-Na) solution. The Drug Group was orally administered phillyrin (300 mg/kg), while Control Group was given an equal amount of 0.5% CMC-Na solution by oral gavage. All animals were fasted for 12 hours before the experiment but had free access to water. The experimental protocol has been approved by the institutional Animal Care and Use Committee in Beijing University of Chinese Medicine. All procedures were carried out according to the Guide for the Care and Use of Laboratory Animals of the US National Institutes of Health.

### 2.3. Sample Collection

#### 2.3.1. Plasma Sample Collection

After oral administration, all rats were placed in metabolic cages. Blood samples (about 0.5 ml) were taken from the infraorbital venous plexus of rats at time points of 0.5, 1, 1.5, 2, and 4 h after administration, and the operation was identical for each rat. Blank and experimental samples were obtained from the Control Group and the Drug Group, respectively. All blood samples were placed in a heparin sodium anticoagulated EP tube for 15 minutes and then centrifuged at 3000 rpm (4°C) for 15 minutes to separate plasma. After that, the plasma from the same group was combined into an aggregate and stored at −80°C until use.

#### 2.3.2. Urine Sample Collection

Urine samples (0–24 h) were collected from each rat using a metabolic cage, and each sample was centrifuged at 3000 rpm (4°C) for 15 min to obtain a supernatant. The urine supernatants of each group of rats were mixed and stored at −80°C until use.

#### 2.3.3. Feces Sample Collection

Fecal samples (0–24 h) were obtained from each rat using a metabolic cage, then dried, and ground to a powder. The fecal powders of each group of rats were mixed. Firstly, 0.5 g of fecal powder was dissolved in 70% methanol, and then it was extracted for 30 min by ultrasonic. Finally, fecal samples of rats in the Control Group and the Drug Group were obtained.

### 2.4. Biological Sample Preparation

All biological samples were prepared by precipitating and concentrating proteins and solid residues. Firstly, the SPE cartridges were pretreated by activation with methanol (5 ml) and deionized water (5 ml). Afterward, plasma, urine, and feces samples (1 ml) were added to the SPE cartridges, respectively. Then, the SPE cartridges were eluted with deionized water (5 ml) and methanol (3 ml) in that order, and the methanol eluates were collected. Finally, the eluates were dried with nitrogen at room temperature, and the residue was redissolved in 5% acetonitrile solution (100 *μ*l) and centrifuged at 14000 rpm (4°C) for 15 min. The supernatant obtained by the above method was used for instrumental analysis.

### 2.5. Instruments and Conditions

All biological samples were analyzed using a UHPLC-Q-Exactive mass spectrometer (Thermo Fisher Scientific, MA, USA) equipped with an ESI ion source. Chromatographic separation was performed on a Waters ACQUITY BEH C18 column (2.1 mm × 100 mm × 1.7 *μ*m; Waters Corporation, Milford, MA, USA), and the temperature was set at 25°C. The mobile phase consists of 0.1% formic acid solution (A) and acetonitrile (B), and the linear gradient was set as follows: 0–1 min, 5% B; 1–3 min, 5%–25% B; 3–8 min, 25%–60% B; 8–15 min, 60%–80% B; 15–20 min, 80%–100% B; 20–24 min, 100% B; 24–25 min, 100%–5% B; and 25–30 min, 5% B. The flow rate was 0.3 mL/min and the injection volume was 2 *μ*L.

The optimal operating parameters in negative and positive ion mode were set as follows: electrospray voltage, 4 kV; capillary temperature, 350°C; sheath gas flow rate, 275 kPa; auxiliary gas flow rate, 138 kPa; auxiliary gas temperature, 300°C; and collision energy, 40 eV. Metabolites were detected from *m*/*z* 100–1500 using full-scan mass spectrometry with a resolution of 70000. Furthermore, the acquisition mode that relies on the parent ion list- (PIL-) DE was used to obtain the MS^*n*^ phase of the acquired data set.

### 2.6. Data Processing

A Thermo Xcalibur 2.1 workstation (Thermo Scientific, Bremen, Germany) was utilized to acquire and process HR-ESI-MS^1^ and MS^*n*^ data. Based on the established screening templates of metabolites, by searching the metabolites with specific molecular weight to compare the high-resolution extracted ion chromatograms of the Control Group and the Drug Group, the metabolites related to phillyrin were selected. To obtain as many phillyrin metabolite ions as possible, the peaks detected with intensity over 10,000 for negative ion mode and 50,000 for positive ion mode were selected for further structural characterization. Based on the exact mass of the metabolite and the set elemental composition, the formula predictor could accurately calculate the chemical formula of all parent ions. The type and number of atoms were set as follows: C [0–40], H [0–60], O [0–25], S [0–2], N [0–5], and ring double bond (RDB) equivalent value [0–15]. At the same time, MetWorks (Version 1.3) and Mass Frontier (Version 8.0) software (Thermo Scientific, Waltham, MA, USA) were used as auxiliary tools for mass fragmentation behaviors analysis and structural identification.

## 3. Results and Discussion

### 3.1. The Construction of Analysis Strategy

**Figure 1:**
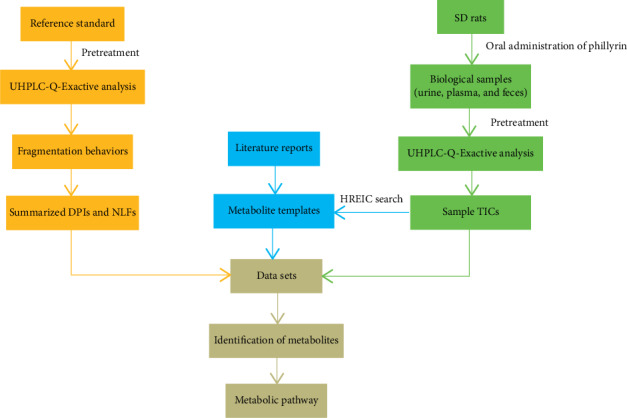
Summary diagram of the developed strategy and methodology.

This study constructed an effective new strategy based on UHPLC-Q-Exactive MS for data acquisition combined with multipath data mining to analyze the metabolism of phillyrin *in vivo*. First of all, the ESI-MS^*n*^ data set of the samples in both positive and negative ion modes were obtained by using the data-dependent scanning (DDS) acquisition method. Secondly, the common metabolites of phillyrin were determined according to literature reports and HREIC search, thereby establishing the screening templates for phillyrin metabolites. Then, by analyzing the mass fragmentation behaviors of the reference standards, the reasonable DPIs and NLFs of phillyrin were summarized. Afterward, based on the chromatographic retention time, the established DPIs and NLFs, and the corresponding calculated ClogP values, the structure of a series of metabolites of phillyrin could be identified. Finally, the metabolic pathway of phillyrin can be inferred from the above metabolic data. The entire research process of this strategy is shown in Figure 1.

### 3.2. Establishment of the Screening Templates for Metabolites

According to literature reports and HREICs search, three compounds (phillyrin, phillygenin, and enterolactone) were selected as primary metabolites, which are often found in the metabolites of phillyrin. After data sorting, in negative ion mode, the metabolites screening templates were set as follows: (1) phillyrin template (*m*/*z* 533.2017); (2) phillygenin template (*m*/*z* 371.1489) and its derivative templates (*m*/*z* 329.1020 for detrimethyl, *m*/*z* 357.1333 for demethylation, *m*/*z* 373.1646 for hydrogenation, and *m*/*z* 387.1438 for oxygenation); (3) enterolactone template (*m*/*z* 297.1121). Based on this effective method, some nondiscoverable metabolites could also be screened out from complex background noise.

### 3.3. Analysis of Mass Fragmentation Behavior of Phillyrin

**Figure 2:**
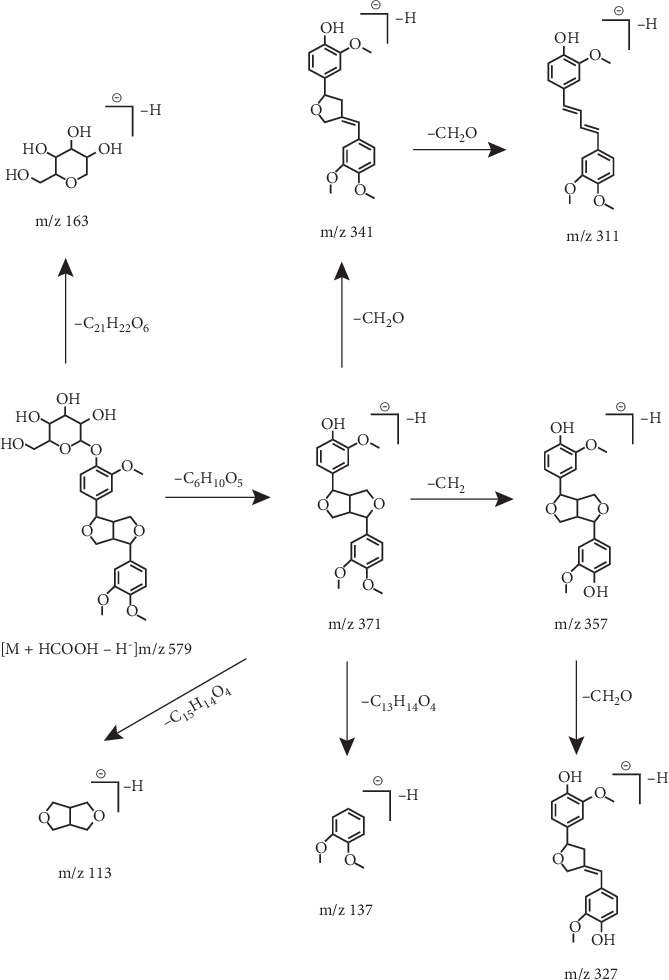
The fragmentation behavior of phillyrin in negative ion mode.

**Figure 3:**
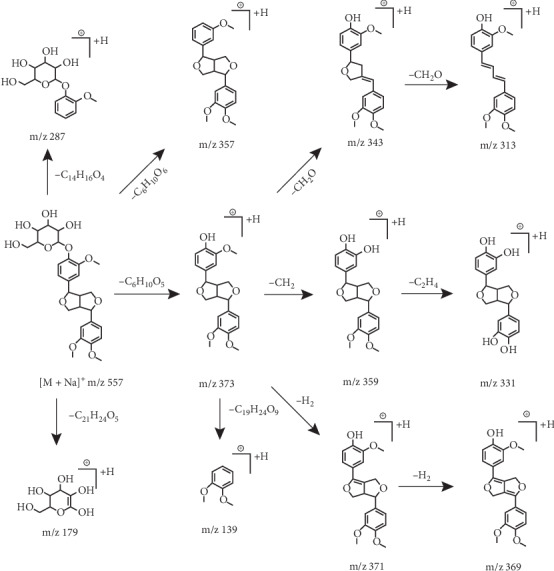
The fragmentation behavior of phillyrin in positive ion mode.

To further explore the ESI-MS^*n*^ fracture behavior of phillyrin, a comprehensive analysis of the standard solution was performed using UHPLC-Q-Exactive MS. For example, phillyrin in negative ion mode could find [M + HCOOH–H]^−^ ion at *m*/*z* 579.2072 in the ESI-MS^1^ spectrum. Due to the absence of the glucose fragment, its characteristic ion peak was found at *m*/*z* 371 of the ESI-MS^2^ spectrum. On this basis, several series of characteristic product ions were retrieved at *m*/*z* 357, *m*/*z* 341, *m*/*z* 327, and *m*/*z* 311 because a series of fragments such as CH_2_, 2CH_2_, CH_2_ + CH_2_O, and 2CH_2_O were successively lost. Compounds with the same parent nucleus will have similar cleavage fragments in the ESI-MS^*n*^ spectrum, so a comprehensive identification of metabolites can be achieved based on regular DPIs and NLFs. For example, the DPI at *m*/*z* 371 was diagnosed due to the absence of a glucose moiety. Thus the presence of DPIs at *m*/*z* 371 or *m*/*z* 371 + X in the ESI-MS^2^ spectrum of the compound provided comparable information for the identification of metabolites. At the same time, the continuous appearance of 14 Da (CH_2_) and 30 Da (CH_2_O) NLFs in the ESI-MS^*n*^ spectrum of phillyrin also provided significant help for the identification of metabolites. The cleavage pathway of phillyrin in negative ion mode is presented in Figure 2. Moreover, the mass fragmentation behaviors of phillyrin in positive ion mode are shown in Figure 3.

### 3.4. Identification of Phillyrin Metabolites in Rats

**Figure 4:**
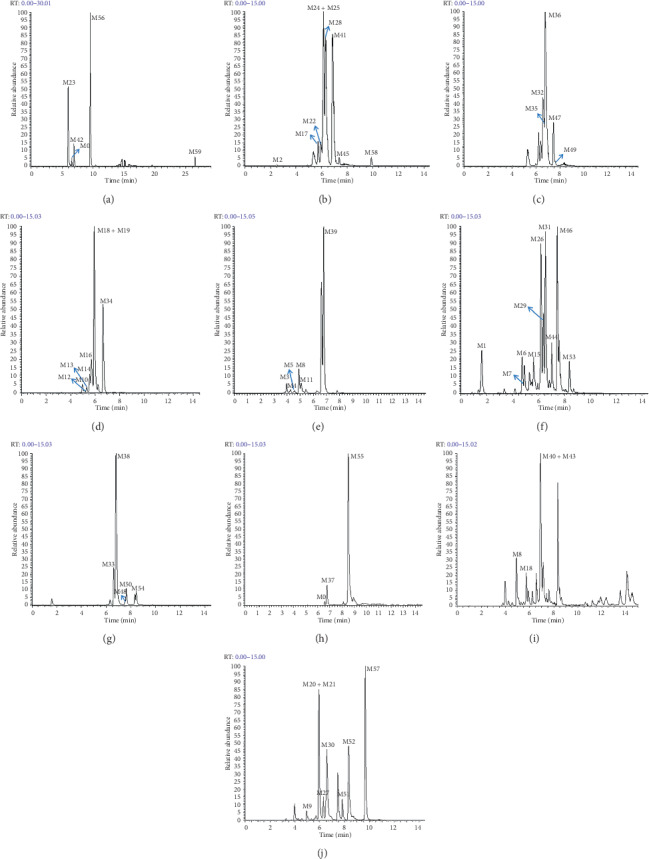
High-resolution extracted ion chromatograms of phillyrin metabolites (A-E) for negative ion mode and (F-J) for positive ion mode): (a) *m*/*z* 339.2035, 417.1179, 451.1057, 507.2225, and 579.2072; (b) *m*/*z* 261.1146, 271.0602, 315.0865, 437.0901, 453.0850, and 519.1498; (c) *m*/*z* 271.1329, 357.1332, 377.0689, 533.1652, and 547.1809; (d) *m*/*z* 329.1021, 333.1327, 345.0968, 359.1490, 375.1074, and 409.0588; (e) *m*/*z* 331.1177, 343.1177, and 347.1126; (f) *m*/*z* 298.0962, 305.1772, 315.1227, 341.1384, 377.1953, and 393.1543; (g) *m*/*z* 231.1404, 331.0811, 355.1541, and 492.1687; (h) *m*/*z* 273.1486, 285.0759, and 557.1993; (i) *m*/*z* 317.0657, 331.1178, 333.1332, and 371.1489; and (j) *m*/*z* 281.1173, 295.0964, 313.1070, 317.1384, 357.1332, 373.1281, and 373.1646.

The total ion chromatograms (TICs) of urine, plasma, and feces samples after oral administration of phillyrin in rats were obtained using UHPLC-Q-Exactive mass spectrometry. A total of 60 metabolites were found in both positive and negative ion modes by processing the data collected from the UHPLC-Q-Exactive instrument. Among them, there were 31 metabolites in positive ion mode and 33 metabolites in negative ion mode. In addition, after the literature search and comparison, 21 metabolites have been found and detected by predecessors [30, 31], while the remaining 39 metabolites have been screened and identified through the established strategy. All relevant mass spectral data are summarized in Table 1, and high-resolution extracted ion chromatograms (HREICs) of all metabolites of phillyrin are shown in Figure 4.

#### 3.4.1. Identification of Metabolites Based on Phillyrin

The metabolite M0 producing [M + HCOOH–H]^−^ ion at *m*/*z* 579.2072 (C_28_H_35_O_13_, 2.32 ppm) was eluted at 6.60 min. In contrast to the elution time and fragmentation behavior of the phillyrin standard, M0 could be accurately inferred as the phillyrin [32, 33].

The retention times of the metabolites M17 and M22 were 5.76 and 5.96 min, respectively, and they showed the same molecular ion at *m*/*z* 519.1498 (C_25_H_27_O_12_, error ≤ ±2.50 ppm). In the ESI-MS^2^ spectrum, the DPI at *m*/*z* 343 was generated due to the loss of 2CH_2_ by the phillygenin. While *m*/*z* 519 was 176 Da larger than the former, it was inferred that *m*/*z* 343 was caused by the absence of the glucuronic acid moiety. Therefore, it was speculated that M17 (Clog*P*, −0.41) and M22 (Clog*P*, −0.39) were demethylated and carboxylated metabolites of phillyrin, but the position of glucuronic acid group was different.

The metabolite M32 was eluted at 6.58 min and showed [M-H]^−^ ion at *m*/*z* 533.1652 (C_26_H_29_O_12_, 2.15 ppm). Similarly, as in the case of M17, the presence of *m*/*z* 175 meant the formation of glucuronic acid. While M32 was 14 Da higher than M17, it was speculated that the phillygenin only had one molecule of CH_2_ removed.

The metabolite M36 with a retention time of 6.76 min showed [M-H]^−^ ion at *m*/*z* 547.1809 (C_27_H_31_O_12_, 2.17 ppm). Similar to the above rule, it was concluded that M36 was the carboxylation product of phillyrin.

Metabolite M56 exhibited [M-H]^−^ ion at *m*/*z* 507.2225 (C_26_H_35_O_10_, 2.28 ppm). It had a neutral loss of 44 Da, 132 Da, and 44 Da in the ESI-MS^2^ spectrum, which caused the appearance of *m*/*z* 463, *m*/*z* 331, and *m*/*z* 287. Among them, the 44 Da NLF can be presumed to be CH_2_O + CH_2_, while the NLF of 132 Da was formed by the loss of CH_2_O from the glucosyl group. Therefore, it was concluded that M56 is the dehydroxylation and hydrogenation product of phillyrin.

#### 3.4.2. Identification of Metabolites Based on Phillygenin

The retention time of the metabolite M44 was 7.05 min, which showed [M + H]^+^ ion at *m*/*z* 305.1772 (C_19_H_21_N_4_, 5.95 ppm). According to the DPI at *m*/*z* 109 and *m*/*z* 93, it could be inferred that two benzene rings of phillygenin were introduced into 2NH_2_ and CH_3_, respectively. Interestingly, *m*/*z* 205 was 28 Da higher than *m*/*z* 177, and *m*/*z* 177 was also 28 Da higher than *m*/*z* 149, so NLF was assumed to be CH=NH by molecular formula and degree of unsaturation. Therefore, M44 was presumed to be the ammoniated product of phillygenin.

The metabolite M2 eluted at 2.49 min showed [M−H]^−^ ion at *m*/*z* 315.0865 (C_17_H_15_O_6_, −8.27 ppm). In the ESI-MS^2^ spectrum, the DPI at *m*/*z* 193 demonstrated that one of the phenyl rings of the phillygenin was introduced into 4OH, and the mother nucleus lost 2CH_2_O. According to the DPI at *m*/*z* 124, 137, and 150, the generation of two double bonds could also be proved.

Metabolite M18 was eluted at 5.92 min and showed [M−H]^−^ ion at *m*/*z* 329.1021 (C_18_H_17_O_6_, 3.33 ppm). The presence of DPI at *m*/*z* 137 and 163 in the ESI-MS^2^ spectrum confirmed the presence of dimethoxyphenyl, while the DPI at *m*/*z*109 speculated that 2OH was introduced on the other benzene ring. However, the substitution sites of the two other hydroxyl groups could not be determined.

The retention time of the metabolite M39 was 6.79 min, which showed [M−H] ions at *m*/*z* 343.1177 (C_19_H_19_O_6_, 3.57 ppm). Similar to the inferred process of M18, after determining the substituents of two benzene rings, it could be concluded that M39 was the product of the loss of 2CH_2_ by phillygenin.

Metabolites M38 and M54 eluted at 6.79 min and 8.44 min, respectively, which showed the same [M + H]^+^ ion at *m*/*z* 355.1541 (C_21_H_23_O_5_, error ≤ ±1.50 ppm). The DPI at *m*/*z* 137, 151 indicated the presence of a bismethoxyphenyl group, while *m*/*z* 189 was 112 Da higher than *m*/*z* 77 (phenyl), inferring the formation of a double bond in the two five-membered rings. Furthermore, *m*/*z* 284 was 30 Da higher than *m*/*z* 254, and it was inferred that both were dehydroxyl and dehydrogenated products of phillygenin, except that the position of the double bond was different.

Metabolite M43 was eluted at 6.93 min and showed [M + H]^+^ ion at *m*/*z* 371.1489 (C_21_H_23_O_6_, −1.12 ppm). It was 2 Da lower than the [M + H]^+^ ion of phillygenin, and combined with DPIs in the ESI-MS^2^ spectrum, M43 could be identified as the dehydrogenation product of phillygenin.

The metabolite M57 was extracted in HREIC at *m*/*z* 373.1281 (C_20_H_21_O_7_, −0.99 ppm) in positive ion mode with a retention time of 9.65 min. The DPI at *m*/*z* 373 was 30 Da higher than *m*/*z* 343, which meant the loss of CH_2_O, while *m*/*z* 343 was 18 Da higher than *m*/*z* 325, indicating that water molecule had been removed. Later, due to the loss of CO NLF, the generation of DPI at *m*/*z* 297 was caused. According to this, it could be inferred that after the dehydrogenation and demethylation reaction of the phillygenin, M57 was formed by oxidation. Still, the position of the hydroxyl group could not be determined.

At 6.92 min, the metabolite M42 was eluted, and the [M−H]^−^ ion was shown at *m*/*z* 451.1057 (C_21_H_23_O_9_S, 3.42 ppm). The DPI at *m*/*z* 371 in the ESI-MS^2^ spectrum can be inferred as phillygenin, and *m*/*z* 451 was 80 Da higher than *m*/*z* 371, which was a great help for us to conclude that M42 is a sulfonated product of phillygenin [30].

The retention time of M33 was 6.59 min, which showed [M + H]^+^ ion at *m*/*z* 492.1687 (C_24_H_30_O_8_NS, −1.35 ppm). The DPI at *m*/*z* 492 was 137 Da higher than *m*/*z* 355, and it was presumed that NLF might be a cysteine-oxygen atom (C_3_H_7_NO_2_S + O). The DPI at *m*/*z* 88 (C_3_H_6_NO_2_) was more potent in demonstrating this so that M33 might be the product of the binding of the phillygenin to cysteine.

The metabolite M58 eluted at 9.90 min produced [M−H]^−^ ions at *m*/*z* 261.1146 (C_15_H_17_O_4_, 4.15 ppm). From the ESI-MS^2^ spectrum, the NLF from *m*/*z* 262 to *m*/*z* 235 might be CH=CH_2_. Similarly, the DPI at *m*/*z* 191 was due to the loss of CH=CH from *m*/*z* 217. Moreover, the reason why the *m*/*z* 235 was 18 Da higher than the *m*/*z* 217 was that one molecule of water was lost. It was presumed that the glucosyl moiety had undergone a reduction reaction, leaving two hydroxyl groups remaining on the ring.

The metabolite M40 with a retention time of 6.87 min showed [M + H]^+^ ion at *m*/*z* 317.0657 (C_16_H_13_O_7_, −1.04 ppm). The DPI at *m*/*z* 133 was 56 Da (2CO) higher than *m*/*z* 77 (phenyl), and *m*/*z* 121 was 44 Da (CO + O) higher than *m*/*z* 77. It was presumed that the original five-membered ring portion has C=O formation. According to the DPI at *m*/*z* 109, it was speculated that 2OH existed in the benzene ring moiety.

The metabolite M47 was eluted at 7.47 min, which had [M−H]^−^ ion at *m*/*z* 357.1332 (C_20_H_21_O_6_, 3.94 ppm). In the ESI-MS^*n*^ spectrum, the DPIs produced by the cleavage of M47, such as *m*/*z* 191, 177, 151, 137, and 123, indicated that the two benzene rings underwent substitution reactions with diverse groups (2OCH_3_, OH + OCH_3_), respectively. At the same time, the NLFs were 30 Da (CH_2_O, from *m*/*z* 357 to *m*/*z* 327) and 54 Da (C_4_H_6_, from *m*/*z* 177 to *m*/*z* 123), it could be inferred that the two benzene rings were mutually connected through a five-membered ring and a double bond. Besides, it was known from the molecular formula that it was difficult to ascertain the substitution site of one OH.

The metabolite M48 was eluted at 7.54 min, and [M + H]^+^ ion was generated at *m*/*z* 331.0181 (C_17_H_15_O_7_, −0.54 ppm). The DPI at *m*/*z* 162 was 69 Da (C_4_H_5_O) higher than *m*/*z* 93 (hydroxybenzene, C_6_H_5_O), thereby confirming that the five-membered ring has undergone dehydrogenation to form a double bond. According to the molecular formula, in addition to the presence of 2OH in both benzene rings, the substitution site of 2OH could not be identified.

The metabolites M8 and M11 with retention times of 4.91 min and 5.08 min, respectively, produced the same [M−H]^−^ ion at *m*/*z* 331.1177 (C_18_H_19_O_6_, error ≤ ±5.00 ppm). In their ESI-MS^*n*^ spectra, the NLFs were 30 Da (from *m*/*z* 333 to *m*/*z* 303) and 28 Da (from *m*/*z* 303 to *m*/*z* 275); this indicated that there was only one five-membered ring in the molecular structure. Further, product ions such as *m*/*z* 109 and *m*/*z* 123 stated the substituent groups (2OH, OH + OCH_3_) on the two benzene rings.

The metabolite M10 was eluted at 4.96 min and showed [M−H]^−^ ion at *m*/*z* 333.1327 (C_18_H_21_O_6_, 4.52 ppm). DPIs generated by the continuous neutral loss of CH_2_O (30 Da) such as *m*/*z* 303 and *m*/*z* 273 indicated that both five-membered rings were introduced into H_2_.

Metabolites M3, M4, and M5 eluted at 3.97 min, 4.27 min, and 4.58 min, respectively, showed the same [M−H]^−^ ion at *m*/*z* 347.1126 (C_18_H_19_O_7_, error ≤ ±4.50 ppm). In the ESI-MS^2^ spectrum, the NLF was 30 Da (from *m*/*z* 347 to *m*/*z* 317), indicating the presence of a five-membered ring in the structural formula. The DPIs which appeared at *m*/*z* 163 and 193 further provided a basis for our inference. According to the DPIs such as *m*/*z* 109, 123, and 137, the substitution of two benzene rings could be inferred. Since the substitution sites of the two hydroxyl groups were difficult to judge, M3, M4, and M5 were isomers.

The retention times of the metabolites M14 and M16 were 5.54 min and 5.69 min, respectively, both of which produced [M−H]^−^ ion at *m*/*z* 409.0588 (C_18_H_17_O_9_S, error ≤ ±3.50 ppm). It was 42 Da lower than M42, presumably owing to the loss of 3CH_2_. DPIs at *m*/*z* 329 and *m*/*z* 80 provided strong evidence for this inference. Thus both M14 and M16 were M42 demethylated products, except that the sulfonic acid groups had different binding positions.

Metabolites M27 and M30 were eluted at 6.31 min and 6.54 min and showed [M + H]^+^ ions at *m*/*z* 357.1332 (C_20_H_21_O_6_, −1.19 ppm) and *m*/*z* 357.1332 (C_20_H_21_O_6_, −2.90 ppm), respectively. In the ESI-MS^2^ spectrum of both, the NLF was 112 Da (C_6_H_8_O_2_, from *m*/*z* 189 to *m*/*z* 77), so a double bond was introduced in the five-membered ring. However, DPI was generated at *m*/*z* 137 in the ESI-MS^*n*^ spectrum of M30, and it was inferred that the bismethoxyphenyl group was present, and M27 did not have this group, so M27 (Clog*P*, 1.14) and M30 (Clog*P*, 1.17) were isomers.

The metabolite M34 with a retention time of 6.65 min showed [M−H]^−^ ions at *m*/*z* 359.1490 (C_20_H_23_O_6_, 3.16 ppm). The DPI at *m*/*z* 344 in the ESI-MS^2^ spectrum generated *m*/*z* 284 by loss of 2CH_2_O, and then the DPIs such as *m*/*z* 192 and *m*/*z* 178 could assume that hydrogenation (+2H_2_) occurred in both five-membered rings and there was one molecular double bond. Further, based on the DPI at *m*/*z* 122, the functional groups on the two benzene rings could be deduced.

The retention times of the metabolites M13 and M19 were 5.30 min and 5.93 min, respectively, and the same [M−H]^−^ ion was produced at *m*/*z* 375.1074 (C_19_H_19_O_8_, error ≤ ± 4.00 ppm). In their ESI-MS^2^ spectra, the NLFs were 28 Da (CO, from *m*/*z* 375 to *m*/*z* 347) and 60 Da (2CH_2_O, from *m*/*z* 347 to *m*/*z* 287), respectively. From this, it was presumed that there was a five-membered ring with a double bond and a dimethoxyphenyl group in the structural formula. Combined with the DPIs such as *m*/*z* 137, 109, and the degree of unsaturation, the structural formula could be inferred. However, the positions of the three hydroxyl groups were still unidentifiable, and M13 and M19 were positional isomers.

The metabolite M6 possessed [M + H]^+^ ion at *m*/*z* 393.1543 (C_20_H_25_O_8_, −8.33 ppm) with a retention time of 4.75 min. In the ESI-MS^2^ spectrum of M6, the DPI at *m*/*z* 393 was 93 Da higher than *m*/*z* 300, presumably due to the neutral loss of hydroxybenzene. At the same time, the DPI at *m*/*z* 300 was 60 Da higher than *m*/*z* 240 and 28 Da higher than *m*/*z* 272, and it was presumed that NLFs were 2CH_2_O (60 Da) and 2CH_2_ (28 Da), respectively. Based on the information obtained, we could conclude that the structural formula of M6 contained hydroxybenzene and dimethoxyphenyl groups, and two of the five-membered rings were hydrogenated to open the ring. The DPIs at *m*/*z* 216, 246, and 361 further confirmed the above judgment, and the substitution sites of three other hydroxyl groups could not be determined.

M24, M28, and M41 showed the same [M−H]^−^ ion at *m*/*z* 437.0901 (C_20_H_21_O_9_S, error ≤ ±3.00 ppm), and they were eluted at 6.14 min, 6.32 min, and 6.88 min, respectively. Their molecular weight was 14 Da lower than that of M42, so they were inferred to be the demethylation products of M42. In the ESI-MS^2^ spectrum, the three metabolites had the same DPIs, such as *m*/*z* 357, 151, 137, and 80, which proved that their benzene rings had identical substituents (2OCH_3_, 2OH). Because the substituents on the benzene rings and the sulfo (-HSO_3_) substitution site were different, M24, M28, and M41 were positional isomers [34].

The retention time of the metabolite M25 showing [M−H]^−^ ion at *m*/*z* 453.0850 (C_20_H_21_O_10_S, 2.62 ppm) was 6.16 min. Because its molecular weight was 16 Da higher than M41, it can be speculated that M25 is the oxidation product of M41. The DPIs retrieved from M25's ESI-MS^*n*^ spectrum, such as *m*/*z* 373, 97, and 80, unexpectedly upheld the above guess.

The metabolite M59 was eluted at 26.48 min and showed [M−H]^−^ ion at *m*/*z* 339.2035 (C_15_H_31_O_8_, −3.67 ppm). Based on the molecular formula and the calculated degree of unsaturation, M59 was presumed to be a compound in which an alkane containing 15 carbon atoms was replaced by eight hydroxyl groups. Also, the DPIs and NLFs shown in the ESI-MS^*n*^ spectrum could prove this conclusion.

The metabolite M52 was detected at 8.34 min, which produced [M + H]^+^ ion at *m*/*z* 373.1646 (C_21_H_25_O_6_, −3.47 ppm). In the ESI-MS^*n*^ spectrum of M52, through various information, it could be undoubtedly identified as the parent compound of phillygenin [31, 35, 36].

The metabolite M23 produced [M−H]^−^ ion at *m*/*z* 417.1179 (C_21_H_21_O_9_, 3.67 ppm), and its retention time was 5.98 min. In the ESI-MS^2^ spectrum, the NLF was 176 Da (from *m*/*z* 417 to *m*/*z* 241), and the unsaturated glucuronide group was presumed to be lost, and the DPI at *m*/*z* 175 confirmed this. The DPIs at *m*/*z* 121 and *m*/*z* 103 indicated that the benzene ring was substituted by two methyl groups, so the substitution position of one methyl group could not be determined.

The metabolite M7 was eluted at 4.84 min and showed [M + H]^+^ ion at *m*/*z* 377.1953 (C_16_H_25_O_10_, 1.56 ppm). In the ESI-MS^2^ spectrum, NLF was 134 Da (from *m*/*z* 377 to *m*/*z* 243), and it was presumed to be a derivative of glucose (C_5_H_10_O_4_). According to the DPIs at *m*/*z* 172 and *m*/*z* 72, the substitution position of four hydroxyl groups could be inferred, of which three hydroxyl groups reacted with the benzene ring, and the other hydroxyl group was substituted on the alkane.

#### 3.4.3. Identification of Metabolites Based on Enterolactone

The metabolite M50 was eluted at 7.63 min, and the [M + H]^+^ ion was displayed at *m*/*z* 231.1140 (C_16_H_23_O, −0.35 ppm). From the DPIs in the ESI-MS^2^ spectrum of M50, such as *m*/*z* 135, 107, and 93, it could be inferred that a hydroxy group was substituted on the benzene ring. Then it could be speculated that another benzene ring had undergone an additional reaction, which itself had been introduced with four hydrogen atoms.

The metabolite M45 produced [M + H]^+^ ion at *m*/*z* 273.0759 (C_15_H_13_O_5_, −0.70 ppm) with a retention time of 7.36 min. In the ESI-MS^2^ spectrum, the DPIs at *m*/*z* 123 and *m*/*z* 95 indicated the substitution of two benzene rings (OH + OCH_3_, OH). At the same time, the NLF was 28 Da (from *m*/*z* 151 to *m*/*z* 123 and from *m*/*z* 255 to *m*/*z* 227), and it was speculated that there were two ketone groups between the two benzene rings.

The metabolite M51 was eluted at 7.83 min and showed [M + H]^+^ ion at *m*/*z* 281.1173 (C_18_H_17_O_3_, −2.07 ppm). In the ESI-MS^*n*^ spectrum, the DPIs at *m*/*z* 263, 235, 133, and 107 fully demonstrated the double bond formation in the two 5-membered ring structures. Besides, the NLF was 16 Da (from *m*/*z* 149 to *m*/*z* 133), indicating that there was still a hydroxyl group whose substitution site was unknown.

The metabolite M37 with a retention time of 6.77 min showed [M + H]^+^ ion at *m*/*z* 285.0759 (C_16_H_13_O_5_, −1.30 ppm). Based on the DPIs located in the ESI-MS^2^ spectrum, such as *m*/*z* 161 and *m*/*z* 85, it can be inferred that three consecutive keto groups were present. Meanwhile, the continuous loss of O atom (16 Da) indicated that there are two hydroxyl groups in the other benzene ring.

The metabolite M20, which was eluted at 5.93 min, had [M + H]^+^ ion at *m*/*z* 295.0964 (C_18_H_15_O_4_, −1.51 ppm). According to the continuous neutral loss of CO (28 Da) (from *m*/*z* 277 to *m*/*z* 249 and from *m*/*z* 249 to *m*/*z* 221) and the DPI at *m*/*z* 186, it could be presumed that there were two double bonds in the two five-membered ring structure. Furthermore, the continuous loss of water molecules (18 Da) further proved the existence of dihydroxyphenyl.

The retention time of the metabolite M1 was 1.61 min, which showed [M + H]^+^ ion at *m*/*z* 298.0962 (C_19_H_12_ON_3_, −2.81 ppm). In the ESI-MS^2^ spectrum of M1, the NLFs were 26 Da (from *m*/*z* 189 to *m*/*z* 163 to *m*/*z* 137), and it was presumably caused by the continuous loss of CN. What is more, the substitution site where a hydroxyl group should also exist in the structural formula could not be determined. Therefore, there were three cyano groups and one hydroxyl group in the structural formula.

The metabolite M21 was eluted at 5.94 min and showed [M + H]^+^ ion at *m*/*z* 313.1070 (C_18_H_17_O_5_, −1.73 ppm). According to the DPIs in the ESI-MS^2^ spectrum, such as *m*/*z* 277, 249, 123, 161, and 173, could be presumed the substitution of two benzene rings (OH + OCH_3_, OH) could be presumed and the existence of a five-membered ring and two double bonds between them. Also, according to the molecular formula, it was known that there was still one hydroxyl substitution site that could not be identified.

Metabolite M53 has a retention time of 8.39 min, which had [M + H]^+^ ion at *m*/*z* 315.1227 (C_18_H_19_O_5_, −9.27 ppm). In the ESI-MS^2^ spectrum of M53, the DPIs at *m*/*z* 93, 109, 167, and 189 could fully clarify the substituents (2OH, OH) on the two benzene rings. At the same time, the NLF was 60 Da (from *m*/*z* 315 to *m*/*z* 255), which was presumed to be due to the continuous loss of CH_2_O, so there were two five-membered rings in the structural formula.

The metabolite M9 was eluted at 4.95 min, and it showed [M + H]^+^ ion at *m*/*z* 317.1384 (C_18_H_21_O_5_, 0.16 ppm). Analogous to the above, based on the DPIs in the ESI-MS_2_ spectrum, such as *m*/*z* 123, 149, 151, 163, and 175, the substituents (OH + OCH_3_) on the two benzene rings were presumed. It was inferred from the degree of unsaturation that the linear alkane between the two benzene rings undergone a dehydrogenation reaction to form a double bond. At the same time, it could be known from the molecular formula that another OH substitution site could not be determined.

The metabolites M15, M26, M29, M31, and M46 were eluted at 5.62, 6.21, 6.38, 6.55, and 7.44 min, respectively, and they all showed the same [M + H]^+^ ion at *m*/*z* 341.1384 (C_20_H_21_O_5_, error ≤ ±1.50 ppm). Among them, in the ESI-MS^2^ spectra of M15, M26, and M29, DPIs at *m*/*z* 137 and *m*/*z* 187 indicated the presence of dimethoxyphenyl and methoxyphenyl. At the same time, NLFs were 28 Da (from *m*/*z* 291 to *m*/*z* 263) and 70 Da (from *m*/*z* 341 to *m*/*z* 271), which proved that there were a five-membered ring and two double bonds between the two benzene ring structures. Additionally, it was difficult to determine the position of one hydroxyl group. Therefore, it was inferred that the three metabolites were isomers because the substitution positions of methoxy and hydroxyl groups were different. However, in the ESI-MS^*n*^ spectra of M31 and M46, the substituents (OH + OCH_3_, OCH_3_) on the two benzene rings could be presumed based on the DPIs at *m*/*z* 123, 151, and 161. Meanwhile, the NLFs were 30 Da (CH_2_O, from *m*/*z* 341 to *m*/*z* 311) and 28 Da (CO, from *m*/*z* 311 to *m*/*z* 283), and the existence of two five-membered rings and the formation of a double bond were further deduced. Because the position of the methoxy group was difficult to determine, M31 and M46 were positional isomers.

The retention time of the metabolite M12 was 5.15 min, and it had [M−H]^−^ ion at *m*/*z* 345.0969 (C_18_H_17_O_7_, 4.20 ppm). DPIs in the ESI-MS^2^ spectrum, such as *m*/*z* 109 and 137, revealed the structure of two benzene rings (2OH, 2OH). The neutral loss of 60 Da (2CH_2_O) resulted in the formation of DPI at *m*/*z* 285, indicating that the two five-membered rings remained unchanged. Also, there was still no known substitution site for a hydroxyl group, so M12 was a demethylation and oxidation product of phillygenin.

The metabolite M35 was eluted at 6.69 min and produced [M−H]^−^ ion at *m*/*z* 377.0689 (C_18_H_17_O_7_S, 3.69 ppm). In the ESI-MS^2^ spectrum, the NLF was 81 Da (HSO_3_), which was presumably due to the neutral loss of the sulfo group. At the same time, the DPIs at *m*/*z* 107 and *m*/*z* 189 revealed the existence of the substituents on the two benzene rings (2OCH_3_, 2OH) and the presence of two double bonds between the benzene rings.

The metabolite M49, which was eluted at 7.61 min, showed [M−H]^−^ ion at *m*/*z* 271.1329 (C_14_H_23_O_5_, 4.68 ppm). In the ESI-MS^*n*^ spectrum, the NLF was 60 Da (from *m*/*z* 271 to *m*/*z* 211), and it was inferred that the two five-membered rings undergo hydrogenation (+H4) to open the ring. The DPIs at *m*/*z* 151 and *m*/*z* 165 proved that the benzene ring had an addition reaction (H_2_) and was replaced by 2OCH_3_. Another OH substitution site was challenging to identify.

The metabolite M55 with a retention time of 8.49 min showed [M + H]^+^ ion at *m*/*z* 273.1486 (C_17_H_21_O_3_, 0.80 ppm). The DPIs in the ESI-MS^2^ spectrum, such as *m*/*z* 179, 137, and 95, clarified the structure of the two benzene rings (OH + OCH_3_, OH). Based on the established molecular formula, it could be inferred that the fabric between two benzene rings was a linear alkane containing four carbon atoms.

### 3.5. Proposed Metabolic Pathways of Phillyrin

**Figure 5:**
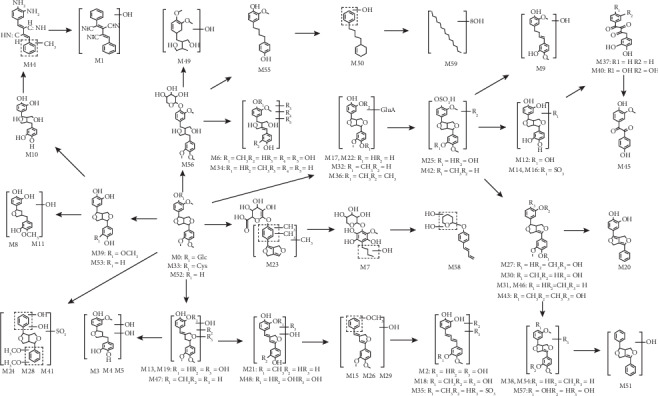
The proposed phillyrin metabolic patterns *in vivo*.

In our study, a total of 60 metabolites (including the prototype compound) were detected and identified in rats that were orally administered phillyrin. The proposed metabolic pathways of forsythiaside are shown in Figure 5. The main biological reactions of phillyrin in rats included the following types, such as hydrogenation, methylation, hydroxylation, glucuronidation, sulfonation, carbonylation, ammoniation, dehydrogenation, demethylation, and ring cleavage and their composite reactions. Also, some extraordinary products had been discovered, such as carbon-nitrogen unsaturated bonds formed in M1 and M44. Moreover, the metabolites M49, M50, and M59, which were generated by hydrogenation or cleavage of benzene, had never been reported before.

## 4. Conclusions

After oral administration of phillyrin to rats, metabolites in plasma, urine, and feces of rats were studied comprehensively. Its innovation lay in the use of a new comprehensive strategy to screen and identify 60 *in vivo* metabolites of phillyrin. Firstly, the combination of UHPLC and Q-Exactive MS overcame many of the shortcomings of traditional triple quadrupole mass spectrometry, and the extremely high resolution could significantly eliminate the interference of the sample matrix. Secondly, the formation of the data set mainly depended on a variety of data mining methods, such as high extracted ion chromatogram (HEIC), diagnostic product ion (DPI), neutral loss filtering (NLF), and isotope pattern filtering (IPF). Finally, accurate qualitative identification of compounds could be achieved based on the precise relative molecular mass of each chromatographic peak in the mass spectrum, the ion fragment information of the secondary mass spectrum, the fragmentation rule of the mass spectrum, and the chromatographic retention time. The results showed that the primary biological reactions of phillyrin *in vivo* included methylation, hydrogenation, sulfonation, glucuronidation, demethylation, dehydrogenation, ring cleavage, and their composite reactions. Among them, the biological activity of some specific metabolites was unknown. In summary, this study provided vital information to the research field of phillyrin metabolism and had important value significance for studying the mechanism of action of phillyrin and monitoring of drug content.

## Figures and Tables

**Table 1:** Summary of phillyrin metabolites in rat urine, plasma, and feces.

Peak	Ionization mode	*t*_R_/min	Formula	Theoretical Mass *m*/*z*	Experimental Mass *m*/*z*	Error (ppm)	MS/MS fragment ions	Identification	U	P	F	Reported/New
M0	N	6.60	C_28_H_35_O_13_	579.2072	579.2086	2.32	MS^2^(579):371(100), 356(31), 372(31), 357(20)341(19), 327(17), 113(8), 122(6), 311(6), 137(4)	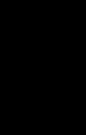	+	+	+	Reported
	P	6.60	C_27_H_34_O_11_Na	557.1993	557.1988	−0.97	MS^2^(557):557(100), 373(56), 357(41), 343(37)371(26), 287(24), 359(18), 369(13), 313(10), 331(7), 179(6), 139(5)		+	+	+	
M1	P	1.61	C_19_H_12_ON_3_	298.0962	298.0967	−2.81	MS^2^(298):136(100), 61(7), 137(5), 145(5), 97(4), 163(3)	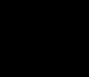	+	−	−	New
M2	N	2.49	C_17_H_15_O_6_	315.0865	315.0837	−8.27	MS^2^(315):150(100), 193(58), 283(49), 151(42)137(29), 182(28), 225(24), 183(23), 192(13), 124(12)	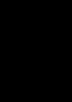	+	−	−	New
M3	N	3.97	C_18_H_19_O_7_	347.1126	347.1138	3.66	MS^2^(347):163(100), 137(41), 165(39), 123(38)151(37), 181(28), 347(18), 193(17), 175(15), 161(10)	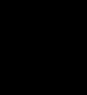	−	−	+	New
M4	N	4.27	C_18_H_19_O_7_	347.1126	347.1141	4.44	MS^2^(347):137(100), 317(26), 347(25), 209(18)139(16), 123(15), 151(13), 163(12), 191(12), 109(10)	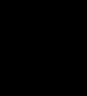	−	−	+	New
M5	N	4.58	C_18_H_19_O_7_	347.1126	347.1140	4.18	MS^2^(347):123(100), 317(94), 193(52), 163(40)165(40), 137(39), 151(28), 318(20), 181(18), 299(17), 347(13), 139(12)	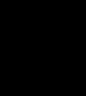	−	−	+	New
M6	P	4.75	C_20_H_25_O_8_	393.1543	393.1511	−8.33	MS^2^(393):300(100), 216(95), 240(19), 301(18)393(14), 217(12)	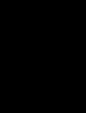	+	−	−	New
M7	P	4.84	C_16_H_25_O_10_	377.1953	377.1448	1.56	MS^2^(377):243(100), 377(50), 244(14), 378(12)99(9), 69(7), 359(6), 172(2), 73(2)	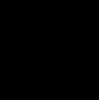	+	−	−	New
M8	N	4.91	C_18_H_19_O_6_	331.1177	331.1190	4.24	MS^2^(331):331(100), 301(86), 123(41), 333(34)332(23), 302(20), 153(18), 177(14), 275(14), 303(9), 109(6)	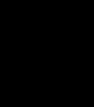	−	−	+	Reported
	P	4.92	C_18_H_21_O_6_	333.1332	333.1330	−0.71	MS^2^(333):123(100), 297(27), 161(19), 149(19)137(18), 175(13), 279(11), 187(11)					
M9	P	4.95	C_18_H_21_O_5_	317.1384	317.1384	0.16	MS^2^(317):123(100), 175(73), 149(31), 163(19)299(19), 137(16), 193(4), 133(3)	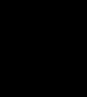	−	−	+	New
M10	N	4.96	C_18_H_21_O_6_	333.1327	333.1348	4.52	MS^2^(333):333(100), 275(28), 334(22), 123(20)303(13), 285(8), 273(3)	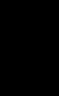	−	−	+	Reported
M11	N	5.08	C_18_H_19_O_6_	331.1177	331.1191	4.61	MS^2^(331):331(100), 301(42), 333(32), 123(23)332(22), 303(3), 109(3)	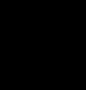	−	−	+	Reported
M12	N	5.15	C_18_H_17_O_7_	345.0968	345.0983	4.20	MS^2^(345):285(100), 345(42), 286(18), 315(16)109(14), 175(14), 137(12), 346(11)	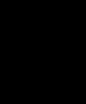	−	−	+	Reported
M13	N	5.30	C_19_H_19_O_8_	375.1074	375.1088	3.70	MS^2^(375):329(100), 330(22), 331(2), 287(1), 137(1), 347(1)	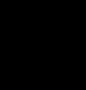	−	−	+	New
M14	N	5.54	C_18_H_17_O_9_S	409.0588	409.0601	3.28	MS^2^(409):329(100), 409(57), 137(30), 330(22)80(15), 410(14)	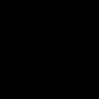	−	−	+	New
M15	P	5.62	C_20_H_21_O_5_	341.1384	341.1381	−0.76	MS^2^(341):137(100), 291(65), 271(61), 163(45), 263(26), 187(25), 323(22), 175(19), 283(17), 153(14), 308(14), 217(14), 279(11)	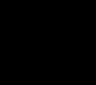	+	−	+	New
M16	N	5.69	C_18_H_17_O_9_S	409.0588	409.0602	3.35	MS^2^(409):329(100), 409(61), 137(31), 330(23), 80(17), 410(15)	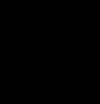	−	−	+	New
M17	N	5.76	C_25_H_27_O_12_	519.1498	519.1508	2.04	MS^2^(519):113(100), 343(72), 85(46), 59(26), 99(19), 75(19), 95(18), 175(17), 344(15), 71(15), 117(14), 519(13)	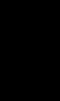	+	−	−	New
M18	N	5.92	C_18_H_17_O_6_	329.1021	329.1031	3.33	MS^2^(329):137(100), 329(19), 138(8), 159(7), 109(6), 163(1)	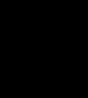	−	−	+	New
	P	5.74	C_18_H_19_O_6_	331.1178	331.1177	0.29	MS^2^(329):149(100), 123(21), 295(13), 150(10)165(10)					
M19	N	5.93	C_19_H_19_O_8_	375.1074	375.1085	2.79	MS^2^(375):329(100), 137(63), 330(22), 189(6), 159(5), 109(4), 123(2)	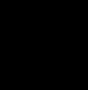	−	−	+	New
M20	P	5.93	C_18_H_15_O_4_	295.0964	295.0960	−1.51	MS^2^(295):277(100), 249(54), 295(48), 241(27)231(24), 259(19), 185(17), 213(14), 280(12), 250(11), 221(5), 186(4)	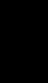	−	−	+	New
M21	P	5.94	C_18_H_17_O_5_	313.1070	313.1065	−1.73	MS^2^(313):123(100), 277(98), 243(69), 249(48)173(44), 225(36), 295(34), 161(23), 175(16), 231(14), 283(13), 265(12), 250(12)	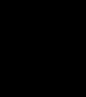	−	−	+	New
M22	N	5.96	C_25_H_27_O_12_	519.1498	519.1508	2.15	MS^2^(301):113(100), 343(85), 85(41), 151(22), 59(21), 95(20), 344(20), 99(19), 75(17), 175(16), 71(16), 117(11), 97(10)	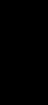	+	−	−	New
M23	N	5.98	C_21_H_21_O_9_	417.1179	417.1195	3.67	MS^2^(417):113(100), 85(48), 241(40), 59(27), 99(25), 75(20), 95(19), 121(17), 117(11), 71(10), 175(9)	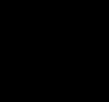	+	+	−	New
M24	N	6.14	C_20_H_21_O_9_S	437.0901	437.0912	2.54	MS^2^(437):151(100), 357(71), 80(66), 437(55), 358(18), 438(16), 342(12)	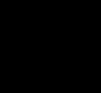	+	+	−	Reported
M25	N	6.16	C_20_H_21_O_10_S	453.0850	453.0862	2.62	MS^2^(453):373(100), 374(26), 453(10), 189(8), 97(6), 80(2)	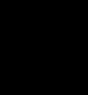	+	−	−	Reported
M26	P	6.21	C_20_H_21_O_5_	341.1384	341.1379	−1.29	MS^2^(341):137(100), 291(75), 271(54), 187(35)239(31), 263(30), 323(29), 175(22), 189(19), 283(18), 217(15), 279(14), 308(14), 311(10)	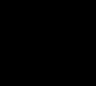	+	−	+	New
M27	P	6.31	C_20_H_21_O_6_	357.1332	357.1328	−1.19	MS^2^(357):123(100), 151(21), 165(19), 357(11), 327(10)	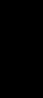	+	−	+	Reported
M28	N	6.32	C_20_H_21_O_9_S	437.0901	437.0913	2.75	MS^2^(437):357(100), 80(75), 437(62), 342(33), 151(3), 137(1)	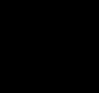	+	+	−	Reported
M29	P	6.38	C_20_H_21_O_5_	341.1384	341.1379	−1.29	MS^2^(341):137(100), 291(65), 271(57), 187(34)239(30), 263(24), 323(22), 283(18), 308(14), 217(14), 272(12), 231(12), 259(10)	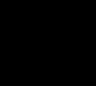	+	−	+	New
M30	P	6.54	C_20_H_21_O_6_	357.1332	357.1322	−2.90	MS^2^(357):151(100), 137(76), 285(66), 201(40)305(36), 254(26), 270(24), 307(23), 297(21), 322(20), 231(18), 338(18), 189(18), 291(17)	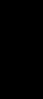	+	−	+	Reported
M31	P	6.55	C_20_H_21_O_5_	341.1384	341.1379	−1.47	MS^2^(341):271(100), 123(96), 292(78), 151(72)201(55), 308(47), 323(44), 305(40), 161(37), 240(35), 256(31), 283(24), 311(22), 277(17)	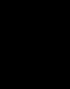	+	−	+	New
M32	N	6.58	C_26_H_29_O_12_	533.1652	533.1665	2.15	MS^2^(533):357(100), 113(84), 85(41), 358(24), 59(22), 71(19), 75(17), 95(16), 99(15), 175(12), 97(12)	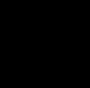	+	−	−	Reported
M33	P	6.59	C_24_H_30_O_8_NS	492.1687	492.1680	−1.35	MS^2^(492):88(100), 151(99), 355(37), 474(35), 323(34), 254(33), 189(32), 336(20), 177(17), 193(15), 369(14), 169(14), 149(13), 181(12)	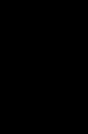	+	−	−	Reported
M34	N	6.65	C_20_H_23_O_6_	359.1490	359.1501	3.16	MS^2^(359):359(100), 360(25), 192(16), 178(14)344(12), 122(8), 284(3)	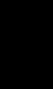	−	−	+	Reported
M35	N	6.69	C_18_H_17_O_7_S	377.0689	377.0703	3.69	MS^2^(377):297(100), 377(54), 298(23), 378(12)107(2), 189(2), 80(1)	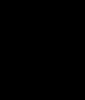	+	−	+	New
M36	N	6.76	C_27_H_31_O_12_	547.1809	547.1822	2.17	MS^2^(547):113(100), 371(60), 85(59), 59(44), 356(38), 95(23), 117(16)87(14), 372(13), 129(13), 357(10), 175(7)	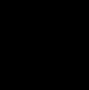	+	−	−	Reported
M37	P	6.77	C_16_H_13_O_5_	285.0759	285.0754	−1.30	MS^2^(285):285(100), 286(20), 85(4), 270(3), 161(1)	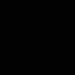	−	+	−	New
M38	P	6.79	C_21_H_23_O_5_	355.1541	355.1535	−1.41	MS^2^(355):137(100), 284(97), 151(74), 306(69)305(57), 201(47), 322(39), 254(37), 270(36), 337(34), 175(30), 297(24), 231(21), 189(19)	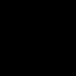	+	−	+	New
M39	N	6.79	C_19_H_19_O_6_	343.1177	343.1188	3.57	MS^2^(343):343(100), 109(71), 137(43), 344(24)151(13), 123(4)	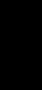	−	−	+	Reported
M40	P	6.87	C_16_H_13_O_7_	317.0657	317.0653	−1.04	MS^2^(317):317(100), 318(21), 302(10), 133(2), 121(1), 109(1)	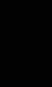	−	−	+	New
M41	N	6.88	C_20_H_21_O_9_S	437.0901	437.0913	2.88	MS^2^(437):357(100), 437(31), 80(19), 97(6), 137(1), 151(1)	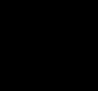	+	+	−	Reported
M42	N	6.92	C_21_H_23_O_9_S	451.1057	451.1073	3.42	MS^2^(451):371(100), 80(81), 451(49), 356(45), 372(24), 452(14), 357(10), 97(9)	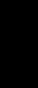	+	+	+	Reported
M43	P	6.93	C_21_H_23_O_6_	371.1489	371.1485	−1.12	MS^2^(371):137(100), 151(15), 165(13), 341(9), 189(5), 353(5)	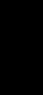	−	−	+	Reported
M44	P	7.05	C_19_H_21_N_4_	305.1772	305.1779	5.95	MS^2^(305):205(100), 223(76), 163(44), 177(31)187(23), 121(20), 149(18), 93(16), 153(16), 206(14), 95(14), 167(14), 224(14), 107(13)	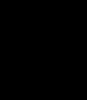	+	−	−	New
M45	N	7.38	C_15_H_11_O_5_	271.0602	271.0615	5.02	MS^2^(271):165(100), 271(56), 272(11), 166(10)	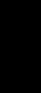	+	−	−	New
	P	7.36	C_15_H_13_O_5_	273.0759	273.0756	−0.70	MS^2^(273):123(100), 273(58), 179(55), 255(36), 151(32), 107(29), 227(15), 274(12), 153(11)					
M46	P	7.44	C_20_H_21_O_5_	341.1384	341.1382	−0.41	MS^2^(341):123(100), 271(92), 292(78), 151(71)270(58), 323(51), 240(38), 161(37), 256(36), 311(34), 283(29), 291(28), 253(26), 277(21)	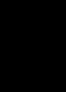	+	−	+	New
M47	N	7.47	C_20_H_21_O_6_	357.1332	357.1347	3.94	MS^2^(357):357(100), 176(25), 358(24), 151(14)342(10), 137(9), 123(8), 177(4), 191(2)	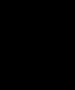	+	−	+	New
M48	P	7.54	C_17_H_15_O_7_	331.0811	331.0811	−0.54	MS^2^(341):331(100), 332(22), 316(8), 315(6), 175(2), 122(2), 162(1), 109(1)	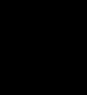	+	−	+	New
M49	N	7.61	C_14_H_23_O_5_	271.1329	271.1553	4.68	MS^2^(271):227(100), 271(30), 209(25), 211(24), 228(16), 151(8), 165(5)	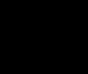	+	−	−	New
M50	P	7.63	C_16_H_23_O	231.1404	231.1743	−0.35	MS^2^(231):135(100), 69(96), 61(57)153(46), 97(41), 107(34), 95(30), 93(29), 83(27), 81(22), 111(22), 109(16), 111(13), 57(10), 136(10)	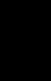	+	−	−	New
M51	P	7.83	C_18_H_17_O_3_	281.1173	281.1166	−2.07	MS^2^(281):133(100), 263(20), 281(11), 134(10), 149(10), 245(6), 107(6), 235(2)	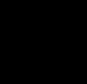	−	−	+	Reported
M52	P	8.34	C_21_H_25_O_6_	373.1646	373.1633	−3.47	MS^2^(373):355(100), 159(51), 161(45), 173(28)337(24), 137(18), 95(17), 121(17), 175(16), 145(16), 149(16), 109(16), 207(15)	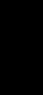	−	−	+	Reported
M53	P	8.39	C_18_H_19_O_5_	315.1227	315.1198	−9.27	MS^2^(315):167(100), 189(49), 315(41), 85(38), 109(12), 168(10), 255(4)	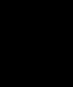	+	−	−	Reported
M54	P	8.44	C_21_H_23_O_5_	355.1541	355.1537	−0.96	MS^2^(355):284(100), 137(96), 151(82), 201(50)337(38), 175(37), 270(36), 297(35), 254(33), 322(32), 189(23), 179(22), 231(20), 310(19)	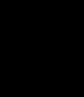	+	−	+	New
M55	P	8.49	C_17_H_21_O_3_	273.1486	273.1487	0.80	MS^2^(273):273(100), 274(14), 117(11), 228(6), 137(6), 229(4), 95(2), 179(1)	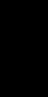	−	+	−	New
M56	N	9.55	C_26_H_35_O_10_	507.2225	507.2236	2.28	MS^2^(507):287(100), 331(34), 113(29), 288(22)507(21), 75(17), 463(14)	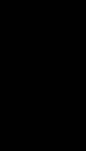	−	+	−	New
M57	P	9.65	C_20_H_21_O_7_	373.1281	373.1278	−0.99	MS^2^(373):343(100), 373(70), 115(62), 358(42)344(21), 374(17), 325(8), 141(2), 297(2)	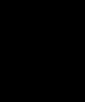	−	+	+	New
M58	N	9.90	C_15_H_17_O_4_	261.1146	261.1132	4.15	MS^2^(261):261(100), 235(70), 217(59), 262(20)236(11)	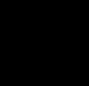	+	+	−	New
M59	N	26.48	C_15_H_31_O_8_	339.2035	339.2001	−3.67	MS^2^(339):339(100), 330(23), 341(3), 62(1)	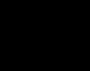	−	+	−	New

Note: *t*_R_: retention time; U: urine; P: plasma; F: feces; +: detected; −: undetected.

## Data Availability

The data used to support the finding of this study are available from the corresponding author upon request.
